# Experiment and Experience

**Published:** 1893-04-01

**Authors:** 


// The Hospital

AN INSTITUTIONAL JOURNAL OF

Science, Medicine, Nursing, and Philanthropy.

*For Hospitals, Asylums, Infirmaries, Dispensaries, Medical Practitioners, Students, Nurses, and the Charitable Public.*

VOL. XIV.—No. 340.] APRIL 1, 1893.

### EXPERIMENT AND EXPERIENCE.

IN his bitter attack upon Jenner and vaccination, Dr. Charles Creighton differentiates sharply between "experiment" and "experience" in medicine. The distinction is just and necessary, and especially necessary at the present time, when by almost universal consent experiment is crowned with olive and bay whilst experience walks meekly along the road uncongratulated and almost denied the right to speak or live. There are tides in the affairs of physic as in the affairs of men, and the weaker spirits, who always constitute the majority, infallibly go with the tides. But the strong, the resolute, and the clear-sighted insist upon their right to be independent of tides, and to apply the best intellectual powers they possess to the formation of judgments which express the fullest measure of truth; of judgments which shall continue to stand when the tides of stormy periods are quiet and disturb no more. Physic, like law and theology, demands patient brains as well as crude experiments.

Bacon twitted the physicians and physiologists of his time with their neglect of experimentation. He was most assuredly right. Experimentation is the life and soul of every practical art, and beyond and above all things physic is a practical art. To experiment we owe not merely the knowledge of the circulation of the blood, of the ligation of arteries, of the cell and the microbial pathologies, and of antiseptic medicine, but almost every certain and solid advance which modern physic has made. Most true; but not to experiment alone; to experiment working side by side with philosophic reasoning; to experiment submitting her work with good or bad grace to the wider test of prolonged experience. It is logic and experience, but especially experience, which in the long run has pronounced upon the work of experiment the sentence of approval or condemnation.

Experimenters are of necessity few; the students of the University of Experience are, equally of necessity, many. The few despise the many, which, however, is of little consequence. What is of consequence is that the many acquiesce in their humiliation, and actually despise one another and themselves. This is a serious misfortune, not only for the many, but still more for medicine as a science and an art. What is the leading object of experimentation? Is it to create a new world? Is it not rather to gain full and accurate knowledge of the world as it already exists, and in a subsidiary measure to acquire power over at least a part of the world in order that we may manipulate the facts of nature to our own advantage? There have been enthusiasts who dreamt that the creation of new worlds lay within their powers. Probably there are no such enthusiasts now on the free side of lunatic asylums. The modest and philosophical aim of modern experimenters is to woo and win truth from willing or unwilling Nature.

But if that be the function of experiment, what is the business of experience? It is to take up the baby of so-called truth which the experimenter has brought into the world; to examine it dispassionately; and, carrying it back to Nature under a thousand different conditions, to find out certainly, yea or no, whether she admits the paternity of her so-called offspring, or whether the infant is a mere abortion of the laboratory and of the crude experimenter's brain. The fact is that experiment is the apprentice and experience the master; experiment is the witness, and experience the jury and the judge. We all know what happens when the workman usurps the master's place; when the witness, leaving his witness-box, claims to be judge and jury as well. Confusion is the result; confusion; error; the failure of practice; general dishonour to all concerned.

Experience has meanly submitted to be snuffed out. Only a week or two ago Dr. Goodhart made melancholy complaint that of all the ripe fruit of the late Sir William Gull's unique experience hardly so much as a gooseberry or a hazel nut remained for the use of future generations. A little scientific work, of no great importance, and work which had been done equally well by other hands is the only monument which remains of Sir William Gull's incomparable technical skill and success as a practising physician. Well, but why is this? It is because nobody wanted, or now wants, to hear anything about experience; because nobody has the patience to study the slowly accumulated facts of the bedside; because nobody will make the intellectual effort to understand the work of years, to extract its leading principles, to master them and incorporate them into daily life and work. What of new had Sir William Gull done? Very little! Nobody asked what of true in art and practice he had achieved, because nobody cared to know. And Sir William Gull knew as well as anybody that nobody cared to know. And therefore his pride did not permit him to cast the rare pearls of his rare experience before the swine of an uncomprehending and unappreciative generation. And who shall blame him? Certainly not the present writer!

The great world showed far more intellectual capacity than the medical profession in its estimate of Sir William Gull. It found him serviceable in time of need. It found him possessed of weapons of knowledge and science which he had tried and proved; not mere crude experimentalising and hasty generalisations of the laboratory; but real knowledge, real science, real confidence, real art and skill which built up the temple of health, and made the world wholesomer and happier. The world was surely right! That is science which stands the test of practice; that, and that alone: just as that is courage which, not boasting in the time of peace, boldly faces the enemy on the battle-field, and conquers or dies.

Our present object is to raise up the drooping head of the practising physician, and to win him back to self-respect, and a just self-reliance. Of course he cannot always keep himself fully abreast of every passing whim and fashion of the physiological laboratory, any more than he can always patronise the latest fashion in hats or the newest shape in trousers. But has he not a laboratory of his own, a clinical laboratory? And is not every patient a subject upon which careful experiments are to be made? To the capable physician every prescription is an experiment: every drug administered is a test of truth.

Do we then despise the physiological laboratory, or wish to exalt the clinical at its expense? By no means! We plead for a truly scientific comprehensiveness of mind. We plead for absolute loyalty to medicine as a science of amelioration and as a practical art. The practising physician is the soldier in the thick of the battle, and his courage must be sustained there by every means which can maintain his self-respect, increase his just self-confidence, and minister to his practical skill in his life-and-death conflict. The professor of military tactics is a very worthy person, but he can never take rank with the heroic soldier. Our physiological experimenters are professors of tactics, and we esteem them very highly in that capacity. But the true leader and defender of the realm of medicine is the practising physician, including, of course, the surgeon. On him lies the responsibility of battle, and to him the world will insist upon giving the greatest honour when he proves himself a hero and a conqueror. Of tactics in the shape of physiological experiments we have enough, perhaps at the moment almost more than enough. What we now want is more historical records of actual warfare, more stories of clinical struggles, defeats, and victories. We have among our leaders men of as great skill and as unique experience as the late Sir William Gull. Let us hope that no passing phase of medical thought will influence them to deprive the world of the rare accumulations of proved science and art which it has been their fortune to gather during the admirable practice of a long lifetime.

